# A double-edged sword: Phosphorylation of Ca^2+^ channel CNGC20 fine-tunes plant freezing tolerance

**DOI:** 10.1093/plcell/koae217

**Published:** 2024-07-23

**Authors:** Leiyun Yang

**Affiliations:** Assistant Features Editor, The Plant Cell, American Society of Plant Biologists; Department of Plant Pathology, College of Plant Protection, Nanjing Agricultural University, Key Laboratory of Integrated Management of Crop Diseases and Pests, Ministry of Education, Nanjing, 210095, China; The Key Laboratory of Plant Immunity, Nanjing Agricultural University, Nanjing, 210095, China

Calcium (Ca^2+^) is an important second messenger in many biological processes, including plant development and stress responses (Tian et al. 2020). Ca^2+^-permeable CYCLIC NUCLEOTIDE GATED CHANNEL (CNGC) proteins transport Ca^2+^ into plant cells to relay external signals into the cell and initiate the appropriate responses (Wang et al. 2021). Little is known about how CNGC proteins are regulated in response to cold stress.

To investigate the potential role of Ca^2+^ channels in cold-induced Ca^2+^ influx, **Yue Peng and colleagues (Peng et al. 2024)** screened 35 T-DNA insertion mutants of Ca^2+^ channel-encoding genes and found that the *cngc20* mutant exhibited reduced freezing tolerance compared with the wild-type (WT) plants. Upon cold treatment, *cngc20* mutant plants showed impaired intracellular calcium accumulation, as indicated by weaker activation of the bioluminescent Ca^2+^ reporter apoaequorin. These findings suggest that CNGC20 may promote plant freezing tolerance by controlling cold-induced Ca^2+^ influx.

To understand how CNGC20 is regulated during freezing tolerance, the authors performed liquid chromatography-tandem mass spectrometry (LC-MS/MS) following co-immunoprecipitation (co-IP) using GNGC20-GFP transgenic plants to screen for CNGC20 interactors. This approach identified the receptor-like kinase PLANT PEPTIDE CONTAINING SULFATED TYROSINE 1 RECEPTOR (PSY1R) as a CNGC20 interactor. The loss-of-function *psy1r* mutant displayed lower survival rates than WT under cold stress, suggesting that PSY1R, similar to CNGC20, promotes plant freezing tolerance. Furthermore, CNGC20 phosphorylation gradually increased over time following cold treatment, but this increase was drastically suppressed in the *psy1r* mutant. Consistent with this observation, an in vitro phosphorylation assay demonstrated that PSY1R directly phosphorylated CNGC20. By replacing 5 putative phosphorylation residues of CNGC20 identified from LC-MS/MS with alanine (mimicking nonphosphorylated forms), they found that phosphorylation of CNGC20 by PSY1R was drastically decreased when Thr-61 or Thr-526 was replaced by alanine. Consistently, the phosphorylation of CNGC20^2A^ (mutating both Thr-61 and Thr-526 to Alanine) was considerably reduced compared with WT CNGC20 under cold stress. These data suggest that PSY1R phosphorylates CNGC20 at Thr-61 and Thr-526 in response to cold stress.

Next, the authors examined whether CNGC20 phosphorylation by PSY1R could affect its Ca^2+^ channel activity using 2-electrode voltage clamping assays in *Xenopus oocytes*. Expressing CNGC20 alone in the oocytes triggered Ca^2+^ influx, and coexpressing PSY1R dramatically enhanced this response. However, this enhancement was abolished when CNGC20^2A^ and PSY1R were coexpressed. These data suggest that CNGC20 phosphorylation by PSY1R promotes its Ca^2+^ channel activity.

In the group's co-IP/LC-MS/MS–based screen for CNGC20 interactors, they also identified COLD-RESPONSIVE PROTEIN KINASE 1 (CRPK1), a known negative regulator of freezing tolerance (Liu et al. 2017). Using similar approaches as for PSY1R, the authors found that CNGC20 was also phosphorylated by CRPK1 and subsequently identified 7 putative phosphorylation sites that did not include Thr-61 and Thr-526. While substituting each of these 7 phosphorylation residues individually with alanine did not significantly change the phosphorylation status of CNGC20, mutating all of them simultaneously (CNGC20^7A^) almost completely abolished phosphorylation by CRPK1. Notably, unlike PSY1R, CRPK1 did not affect the Ca^2+^ channel activity of CNGC20 but promoted CNGC20 degradation by phosphorylation. Under prolonged cold stress, CNGC20 was degraded over time, but this degradation was delayed in the *crpk1* mutant. Additionally, CNGC20^7A^ degraded more slowly than CNGC20, indicating that CRPK1-mediated phosphorylation triggers CNGC20 degradation. These results suggest that CRPK1 negatively regulates freezing tolerance by phosphorylating CNGC20, targeting it for degradation under prolonged cold stress.

This study showcases the sophisticated regulation of CNGC20 through phosphorylation by 2 different kinases: PSY1R enhances its Ca^2+^ channel activation, while CRPK1 promotes its protein degradation, thereby fine-tuning Ca^2+^-mediated freezing tolerance in plants (see Figure). It also highlights the significance of post-transcriptional modifications in regulating plasma membrane-localized proteins. It will be interesting to further explore whether other post-transcriptional modifications also regulate CNGCs and to investigate the potential crosstalk among these modifications under various environmental conditions.

**Figure.**
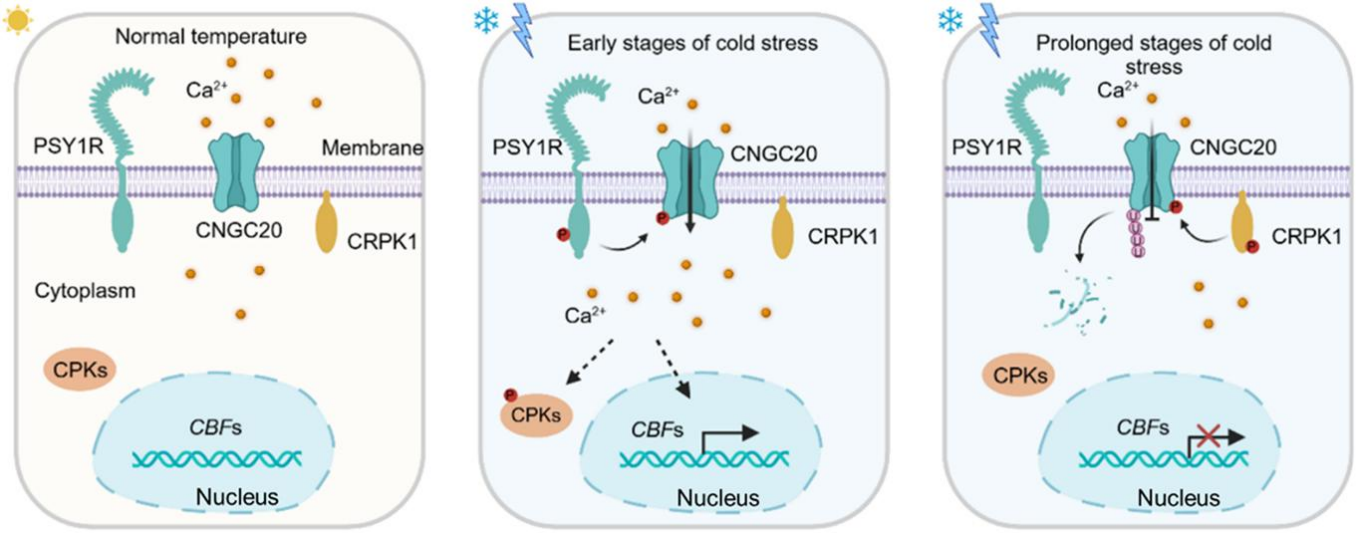
Under normal temperatures, cytosolic Ca^2+^ levels remain at a low level. Upon cold stress, PSY1R rapidly phosphorylates and activates the Ca^2+^ channel CNGC20, resulting in Ca^2+^ influx and subsequent transcriptional activation of cold-responsive genes. Under prolonged cold stress, CNGC20 is phosphorylated by CRPK1 for degradation, leading to a compromised cold response. Adapted from Peng et al. (2024), Figure 7.
